# A room-temperature ultrasonic hydrogen sensor based on a sensitive layer of reduced graphene oxide

**DOI:** 10.1038/s41598-020-80875-0

**Published:** 2021-01-28

**Authors:** Xue-Yu Zhang, Ren-Hao Ma, Ling-Sheng Li, Li Fan, Yue-Tao Yang, Shu-Yi Zhang

**Affiliations:** Lab of Modern Acoustics, Institute of Acoustics, Nanjing University, Nanjing, 210093 China

**Keywords:** Applied physics, Techniques and instrumentation

## Abstract

It is challenging to increase the sensitivity of a hydrogen sensor operating at room temperature due to weak sorption and tiny mass of hydrogen. In this work, an ultrasonic sensor is presented for detecting hydrogen, which is composed of a 128° YX-LiNbO_3_ substrate and a reduced graphene oxide (RGO) sensitive layer with a platinum catalyzer. By optimizing the depositing parameters of RGO and platinum, a considerably high sensitivity is achieved at room temperature. A frequency shift of 308.9 kHz is obtained in 100 ppm hydrogen mixed with argon, and a frequency shift of 24.4 kHz is obtained in 1000 ppm hydrogen mixed in synthetic air. It is demonstrated that in addition to strong sorption of the sensitive layer, the coaction of mass load and conductivity variation is key to high sensitivity of the sensor. By establishing the original conductivity of the sensitive layer within the “conductivity window” for enhancing electrical response, we improve the sensitivity of the ultrasonic sensor, which is available for detecting hydrogen with an extremely low concentration of 5 ppm.

## Introduction

Hydrogen (H_2_) is considered to be a clean and renewable energy source that is promising in various fields as energy battery, chemical production, medical, aerospace and etc. Due to flammability and explosibility, H_2_ must be strictly monitored in the future application. However, H_2_ is difficult to detect because of its colorlessness and odorlessness, and thus, to prevent danger induced by H_2_ leakage, sensors with high sensitivities for detecting early H_2_ leakage with trace concentrations are required^1–5^. The majority of H_2_ sensors work on the basis of sensitive layers for H_2_ sorption. The performances of these sensors are apt to be influenced by the operating temperature, and the sensitivities decrease remarkably with the decrease of temperature due to weak sorption and tiny mass of H_2_. Therefore, it is challenging to increase the sensitivity of a H_2_ sensor operating at room temperature.

Ultrasonic sensors, which are primarily composed of a piezoelectric substrate and a sensitive layer deposited on the substrate, are widely studied for gas sensing owing to distinct advantages of high sensitivities, low detection limits, small bulks, low power dissipation and high integration^6^. When the sensitive layer sorbs the targeted gas, the acoustic velocity and transmission loss of the ultrasonic wave transmitting in the sensor are changed, and by measuring the shift of the central frequency and/or insert loss of the sensor, the targeted gas can be detected. Because ultrasonic sensors work with high operating frequencies from several Mega to Giga hertz, the central frequency and transmission loss are extremely sensitive to the sorbed matters and the sensitivity increases with the operating frequency^7^.

To improve performance, ultrasonic sensors for detecting H_2_ were originally designed to work at high temperatures^8^, in which metallic oxides, as tungsten trioxide (WO_3_)^9^, indium oxide (InO_x_)^10,11^, tin dioxide (SnO_2_)^12^, were found to be efficient for H_2_ sensing. The ultrasonic sensors using sensitive layers based on metallic oxides exhibited high performance at high temperatures of 100–200 °C, in which frequency shifts from 100 kHz to over 700 kHz were obtained in H_2_ with a concentration of 1%^9–12^.

Resembling to other types of H_2_ sensors based on sorption mechanism, the sensitivities of ultrasonic sensors decrease at room temperature because of weak reaction between H_2_ and sensitive layers^13^. Nevertheless, in spite of high sensitivities, heating a H_2_ sensor is an unfavorable way due to explosion danger and energy wastage. Therefore, high-sensitivity hydrogen sensors working at room temperature were given high consideration in the research of ultrasonic sensors. Firstly, H_2_ sensors working at room temperature were tested in nitrogen^14–16^, and a sensitivity of 67 kHz towards 100 ppm H_2_ mixed in N_2_ was obtained based on organometallic conjugated polymers, Pd–DEBP and Pd/Pd–DEBP^16^. It was more challenging to obtain a high sensitivity when a H_2_ sensor works in air because the oxygen in air reacts with H_2_ and reduces the H_2_ molecules adsorbed by the sensitive layer^17–19^. Layered surface acoustic waves, which are apt to be influenced by the mass load induced by the adsorbed gas, were adopted to increase the sensitivities, in which frequency shifts of 27.9 kHz and 34.6 kHz towards 1% H_2_ mixed in air were obtained at room temperature with sensitive layers of ZnO nanorods and CSA synthesized polyaniline nanofibers, respectively^20,21^. Additionally, Rayleigh wave sensors based on Pt modified InO_x_ and WO_3_ sensitive layers exhibited sensitivities of 20 kHz and 72 kHz in 1% H_2_^22,23^. However, it is difficult to further increase the sensitivity of an ultrasonic H_2_ sensor operating in air and at room temperature.

Recently, a new type of material, graphene, attracted great attention due to its unique characteristics^24–26^. It was shown that graphene is also a good sensitive material for H_2_ sensing owing to its large specific surface area, high carrier mobility and low Johnson noise. On the one hand, good electrical properties of graphene were given high consideration and the majority of H_2_ sensors using graphene-like sensitive layers worked on account of the variation of conductivity induced by adsorbed H_2_^5,27–31^. On the other hand, ultrasonic H_2_ sensors using graphene-like sensitive layers were also presented^32–34^. By adopting Pd as a catalyzer, the sensitivity was increased from 5.8 kHz to 30 kHz towards 1% H_2_ mixed in air at room temperature^36^. It can be observed that these ultrasonic H_2_ sensors using graphene-like sensitive layers exhibited comparative sensitivities to their counterparts with the sensitive layers based on metallic oxides and other nano films^20–23^.

In this work, we create an ultrasonic H_2_ sensor composed of a 128° YX-LiNbO_3_ piezoelectric substrate and a graphene-like sensitive layer modified by a Pt catalyzer. The sensitive layer on the basis of the reduced graphene oxide (RGO) is characterized by X-ray diffraction (XRD), Fourier transform infrared spectroscopy (FTIR) and scanning electron microscope (SEM), which demonstrates high performance in H_2_ adsorption. Additionally, owing to the coaction of mass load and conductivity variation induced by the adsorbed H_2_, we achieve an extremely high sensitivity at room temperature by accurately controlling the original conductivity of the RGO/Pt composite sensitive layer. The sensor exhibits a frequency shift of 302.9 kHz towards 100 ppm H_2_ mixed in argon, and in the environment of air, a frequency shift of 24.4 kHz is obtained in 1000 ppm H_2_. Our sensor based on a graphene-like sensitive layer can be available to detect H_2_ with extremely low concentrations below 5 ppm.

## Results

### Fabrication and characterization of the sensitive layer

An oxidation–reduction method is adopted to create graphene-like sensitive layer on the surface of a 128° YX-LiNbO_3_ piezoelectric substrate. First, graphene oxide (GO) is prepared using modified Hummers method^24^, in which graphite powder is oxidized with strong oxidants and oxygen-containing functional groups (OCFGs) are induced into the layered structure of graphite. Then, the layered structure is exfoliated using an ultrasonic cleaner and an ultrasonic cell pulverizer. To remove the OCFGs, hydrothermal reduction method is adopted to reduce the GO^25^, and thus, we obtain a solution of reduced graphene oxide. Finally, we drop the solution on the surface of a 128° YX-LiNbO_3_ substrate with a pair of interdigital transducers and dry it at room temperature. In our experiments, this sedimentation method is more efficient than spin coating or pulling method because the concentration of the RGO solution is low. Using this method, we can rapidly grow sensitive layers on multiple samples. Additionally, it was shown that pure graphene cannot effectively adsorb H_2_^32^, and catalyzers are required to enhance the reaction between the graphene-like sensitive layer and H_2_. In our experiments, we deposit Pt as a catalyzer on the RGO layer via magnetron sputtering method (see “Methods” for details).

**Figure 1:**
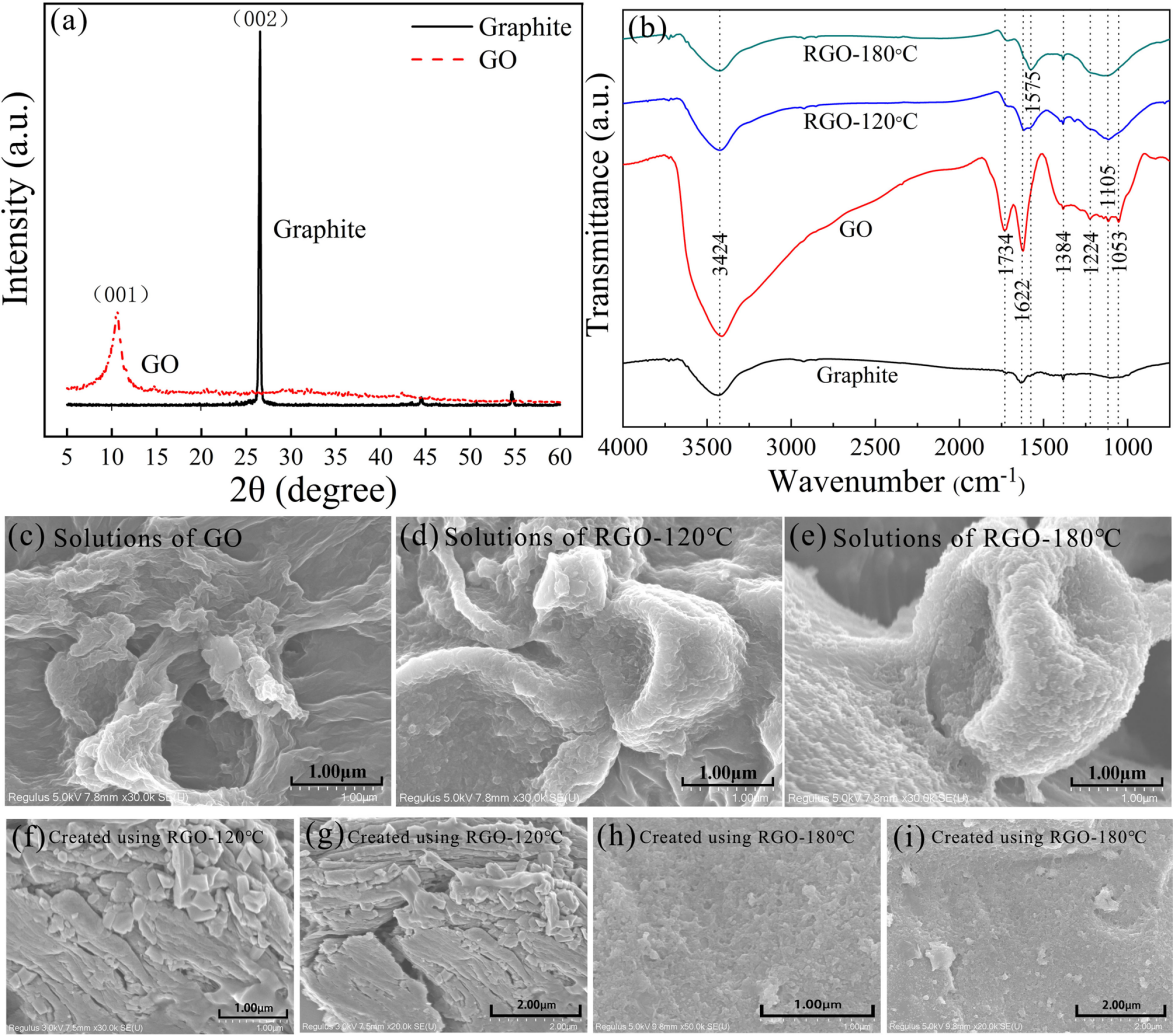
Evaluation of the sensitive layer. (**a**) XRD images for graphite and GO. (**b**) FTIR spectra for graphite, GO, RGO-120 °C and RGO-180 °C. (**c**–**e**) SEM images for (**c**) GO, (**d**) RGO-120 °C and (**e**) RGO-180 °C. (**f**–**i**) SEM images with different resolutions for sensitive layers created on LiNbO_3_ substrates using (**f**,**g**) RGO-120 °C and (**h**,**i**) RGO-180 °C.

Figure 1a shows the images of XRD of graphite and GO obtained by the processes of oxidation and exfoliation. It can be observed that graphite exhibits a high and sharp characteristic diffraction peak at 26.5°, which indicates the 002 crystal plane of graphite. In the XRD image of GO, the diffraction peak shifts to 10.6°, and the peak is much lower and wider than the diffraction peak obtained in graphite. Because the OCFGs increase the distance between the crystal planes, the diffraction peak of GO is shifted to a smaller angle. Furthermore, the OCFGs inserted between layers break the ordered structure of graphite, which results in a lower and wider diffraction peak in GO.

To optimize the RGO obtained by hydrothermal reduction method, we adopted two reaction temperatures, 120 °C and 180 °C, in the reduction. The images of FTIR for different samples, graphite, GO, RGO-120 °C and RGO-180 °C are shown in Fig. 1b. The FTIR spectrum for graphite shows three absorption peaks at 3424 cm^−1^, 1622 cm^−1^ and 1384 cm^−1^, which are induced by the absorbed water molecules in graphite. The absorption peaks arising at 3424 cm^−1^ are related to the stretching vibration of –OH in a water molecule, while the weak peaks at 1384 cm^−1^ and 1622 cm^−1^ originate from the flexural vibration of –OH. For GO, it is observed that the three absorption peaks induced by water molecules (3424 cm^−1^, 1622 cm^−1^ and 1384 cm^−1^) are higher than those obtained in graphite, which demonstrates that GO stores more water than graphite does^35^. Furthermore, different absorption peaks can be observed in the FTIR spectrum for GO, which are induced by the stretching vibration of distinct OCFGs like C=O (1734 cm^−1^), C–O–C (1224 cm^−1^), and C–O (1105 cm^−1^ or 1053 cm^−1^). Thus, it is observed that abundant OCFGs are created in the process of oxidation, which enhances hydrophily of GO.

Comparing the FTIR spectra of RGO and GO indicates that the absorption peaks induced by water molecules and OCFGs are weakened in hydrothermal reduction. Furthermore, the absorption peaks at 1224 cm^−1^, 1105 cm^−1^ and 1053 cm^−1^ even vanish in RGO-180 °C. Thus, it is demonstrated that the OCFGs in GO are removed in the reduction process, and the RGO obtained at a higher reduction temperature possesses less OCFGs. Furthermore, in the FTIR spectra of RGO, an absorption peak arises at 1575 cm^−1^, which cannot be observed in the spectrum of GO. This peak is created by C=C bonds in RGO, demonstrating that the broken π conjugated structure is recovered in the hydrothermal reduction. Additionally, in RGO-180 °C, the absorption peak at 1575 cm^−1^ is much higher than that in RGO-120 °C, indicating better recovery of the π conjugated structure in RGO-180 °C.

To explore the micro-structures of the sensitive layers, the SEM images of different samples are shown in Fig. 1c–i. Figure 1c–e exhibit the SEM images for GO, RGO-120 °C and RGO-180 °C. Compared to RGO samples, the GO sample possesses larger grain sizes and a small number of folds. The RGO samples exhibit agglomerate because the reduction of GO results in the heterogeneity and increases the porosity as a result of exfoliation and rearrangement of layers^36–38^. Additionally, it was shown that a higher degree of reduction of GO resulted in smaller grains, more folds and larger specific surface areas^39^. Thus, comparing Fig. 1f–i shows that the grains of the sensitive layer obtained with the solution of RGO-120 °C are much longer than that created using the solution of RGO-180 °C. The sensitive layer of RGO-180 °C exhibits smaller grain sizes and a large number of folds on the surface, which remarkably increase the porosity and specific surface area, providing abundant adsorption points for Pt and H_2_ molecules. Owing to this feature, the RGO-180 °C sensitive layer exhibits great potentials as a sensitive material for H_2_ sensing.

### H_2_ sensing

**Figure 2:**
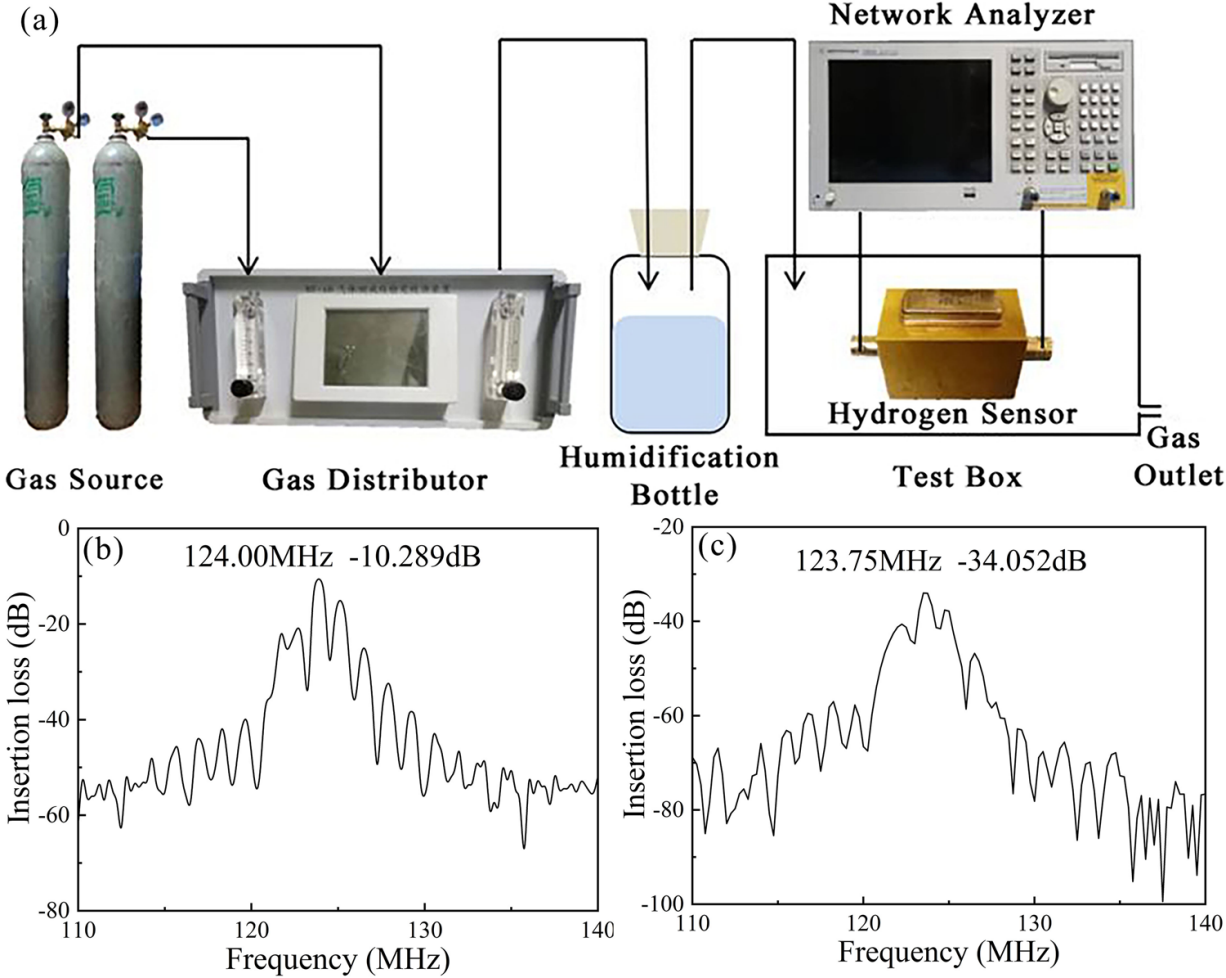
Experimental system. (**a**) Experimental apparatus for H_2_ sensing. (**b**,**c**) Frequency responses of the ultrasonic H_2_ sensor measured (**b**) before and (**c**) after depositing the sensitive layer.

Figure 2a shows our experimental system for H_2_ sensing. To test the performance of our sensor for detecting H_2_ with low concentrations, we use two gas bottles, one contains H_2_ with a concentration of 1000 ppm mixed with a background gas, argon or synthetic air, and the other contains the background gas. The H_2_ with the concentration of 1000 ppm and the background gas are mixed in a gas distributor and the concentration of the H_2_ is lowered further. Then, we have H_2_ with the concentrations from 5 to 1000 ppm. The mixed gas is introduced into a humidification bottle to adjust the humidity. Finally, the gas is tested using our H_2_ sensor in a closed box. Multiple parameters, the central frequency, insert loss and surface electric conductivity, of the sensor are measured by a network analyzer and a 4-probes resistivity measurement system.

Figure 2b,c show the frequency responses of the sample before and after coating the sensitive layer, respectively. The original central frequency and insert loss are 124 MHz and 10.80 dB, which are shifted to 123.75 MHz and 34.05 dB by depositing the RGO/Pt sensitive layer.

**Figure 3:**
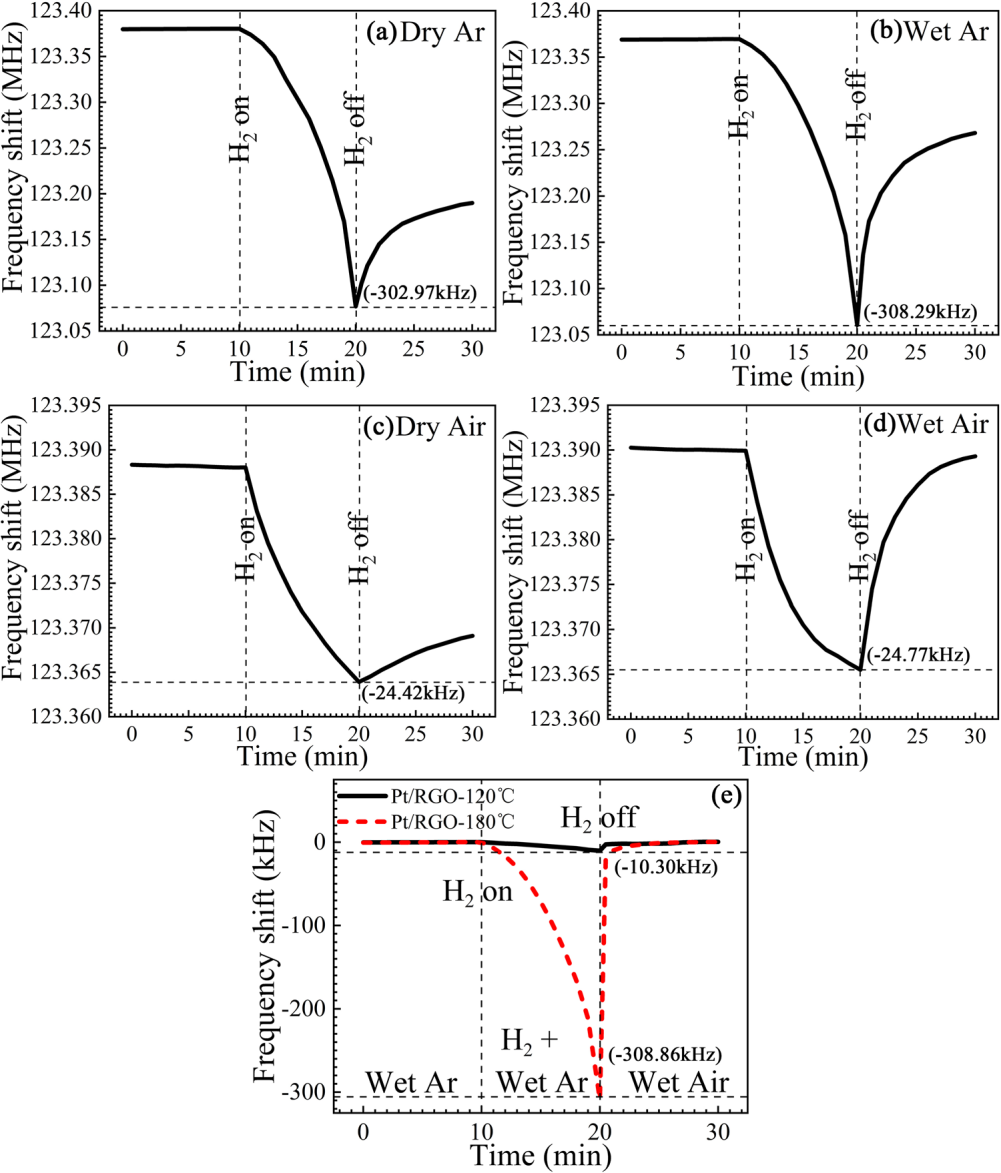
Frequency shift of the sensor towards H_2_. (**a**,**b**) Shifts of the central frequency of the sensor to 100 ppm H_2_ mixed in (**a**) dry argon and (**b**) argon with a relative humidity of 60%. (**c**,**d**) Shifts of the central frequency of the sensor to 1000 ppm H_2_ mixed in (**c**) dry air and (**d**) air with a relative humidity of 60%. (**e**) Shifts of the central frequencies of the sensors with RGO-120 °C and RGO-180 °C sensitive layers to 100 ppm H_2_ mixed in argon with a relative humidity of 60%.

H_2_ sensing experiments are first conducted in argon and the dynamic responses of the sensor are shown in Fig. 3. As shown in Fig. 3a,b, by introducing 100 ppm H_2_ mixed with dry argon, we obtain a frequency shift of 303.5 kHz within 10 min. By increasing the relative humidity of the argon to 60%, we obtain a frequency shift of 308.9 kHz. Furthermore, after 10 min, the central frequency of the sensor continues to decrease rapidly, which demonstrates dramatic H_2_ adsorbability of the sensitive layer. Thus, the sensor exhibits an extremely high sensitivity to H_2_ with a low concentration. Due to the strong chemical adsorption of H_2_ in the RGO/Pt sensitive layer, the central frequency of the sensor returns slowly after we stop the mixed gas flow and introduce pure argon. It can be observed in Fig. 3b that the recovery of the sensor is better in an environment with a high humidity.

Furthermore, we measure the frequency shift of the sensor to H_2_ mixed in synthetic air. As shown in Fig. 3c,d, the sensor exhibits a frequency shift of 24.1 kHz to 1000 ppm H_2_ in dry air, and by increasing the relative humidity to 60%, we obtain a frequency shift of 24.4 kHz. Although the sensitivity of the sensor working in air is lower than that of the sensor operating in argon, our sensor is much more sensitive than those using traditional sensitive layers^20–23^. Resembling to the experiments obtained in argon, in an environment with a high humidity, the sensor recovers faster when H_2_ is exhausted. The performance of the H_2_ sensor is compared to those of the sensors adopting traditional sensitive layers in Table 1.

**Table 1:** Performance of ultrasonic H_2_ sensors.

No	References	Substrate	Sensitive layer	Hydrogen concentration	Frequency shift	Background gas	Working temperature
1	8	YZ-LiNbO_3_	CuPc + Pd	1%	0.2 kHz	Nitrogen	30 °C
2	9	36◦ YX-LiTaO_3_	WO_3_ + Pt	1%	118 kHz	Air	270 °C
			WO_3_ + Au		705 kHz		
3	11	36◦ YX-LiTaO_3_	InOx	1%	515 kHz	Air	290 °C
4	13	ZnO/ 64◦ YX LiNbO_3_	Polyaniline + HCl	1%	3 kHz	Air	Room temperature
			Polyaniline + CSA		14.6 kHz		
5	18	ZnO/ 64◦ YX-LiNbO_3_	polyaniline/WO_3_	1%	7 kHz	Air	Room temperature
6	19	36◦ YX-LiTaO_3_	polyaniline	1%	9.2 kHz	Air	Room temperature
7	22	128◦ YX-LiNbO_3_	WO_3_ + Pt	1%	72 kHz	Air	Room temperature
8	32	36◦ YX-LiTaO_3_	Graphene-like	1%	5.8 kHz	Air	Room temperature
9	33	AlN/Si	Pd-Graphene	1%	30 kHz	Air	Room temperature
10	This work	128◦ YX-LiNbO_3_	Pt-RGO	100 ppm	302.9 kHz	Argon	Room temperature
				1000 ppm	24.4 kHz	Air	

In Fig. 3e, we compare the performances of the sensitive layers fabricated using RGO-120 °C and RGO-180 °C. It is demonstrated that a much higher sensitivity is achieved with the sensitive layer fabricated using RGO-180 °C with a high reduction degree. The high performance of RGO-180 °C is determined by the optimized micro-structure, small grain sizes, high porosity and numerous folds, resulting in a large surface area, which is exhibited in the FTIR and SEM images shown in Fig. 2.

## Discussion

In addition to the high performance of the sensitive layer created using RGO-180 °C, the high sensitivity of our sensor is related to a critical sensing mechanism of ultrasonic gas sensors. Generally, mass load induced by the sorbed matter was considered to be the major factor causing central frequency shift, and thus, the mass sensitivity was defined to be ${S_m} = \mathop {\lim }\limits_{\Delta m \to 0} {{\left( {{{\Delta V} \mathord{\left/ {\vphantom {{\Delta V} V}} \right. \kern-\nulldelimiterspace} V}} \right)} \mathord{\left/ {\vphantom {{\left( {{{\Delta V} \mathord{\left/ {\vphantom {{\Delta V} V}} \right. \kern-\nulldelimiterspace} V}} \right)} {\Delta m}}} \right. \kern-\nulldelimiterspace} {\Delta m}}$^40,41^, where $\Delta m$ is the tiny mass of the sorbed matter and $\Delta V$ is the mass-induced variation of the velocity *V* of the ultrasonic wave transmitting in the sensor. However, it was presented that the central frequency of an ultrasonic sensor is also influenced by the variation of the surface conductivity induced by the sorbed matter, and the conductivity sensitivity was thus defined to be ${S_c} = \mathop {\lim }\limits_{\Delta \sigma \to 0} {{\left( {{{\Delta V} \mathord{\left/ {\vphantom {{\Delta V} V}} \right. \kern-\nulldelimiterspace} V}} \right)} \mathord{\left/ {\vphantom {{\left( {{{\Delta V} \mathord{\left/ {\vphantom {{\Delta V} V}} \right. \kern-\nulldelimiterspace} V}} \right)} {\Delta \sigma }}} \right. \kern-\nulldelimiterspace} {\Delta \sigma }}$^42–44^, indicating the relative velocity variation ${{\Delta V} \mathord{\left/ {\vphantom {{\Delta V} V}} \right. \kern-\nulldelimiterspace} V}$ induced by the conductivity shift $\Delta \sigma$. Especially, in sensors for detecting a light gas, like H_2_, the frequency shift induced by the variation of surface conductivity even overwhelms that caused by the mass load, resulting in an abnormal response that the central frequency increased after the sensor sorbed the targeted gas^42–45^.

**Figure 4:**
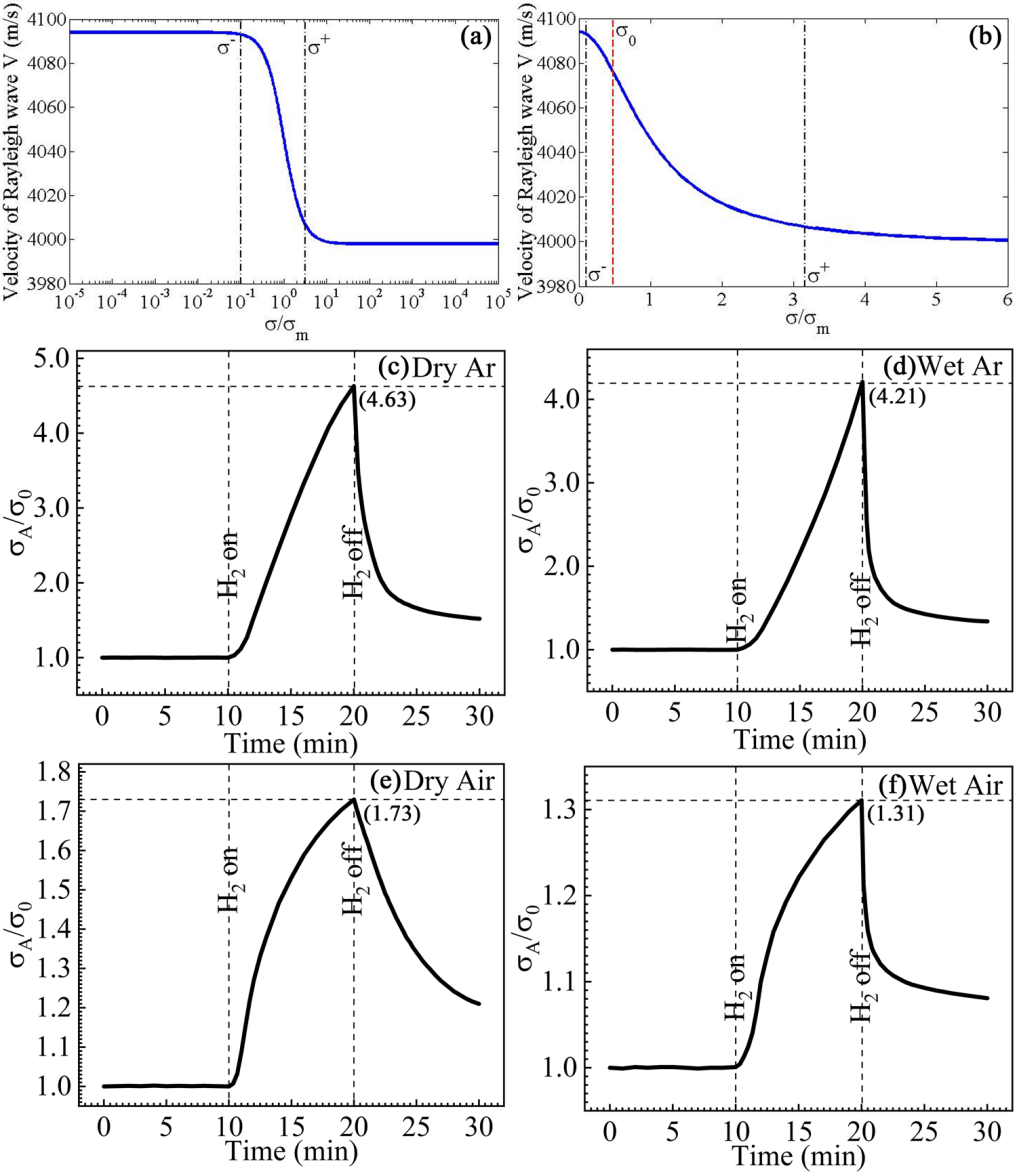
Conductivity variation of the sensor towards H_2_. (**a**,**b**) The velocity *V* of the Rayleigh wave transmitting in the sensor versus the normalized surface conductivity ${\sigma \mathord{\left/ {\vphantom {\sigma {\sigma_m}}} \right. \kern-\nulldelimiterspace} {\sigma_m}}$. It is observed that the Rayleigh wave velocity varies rapidly with the conductivity in a narrow conductivity window, indicating a high conductivity sensitivity. (**c**,**d**) Responses of the conductivity of the sensor to 100 ppm H_2_ mixed in (**c**) dry argon and (**d**) argon with a relative humidity of 60%. (**e**,**f**) Responses of the conductivity of the sensor to 1000 ppm H_2_ mixed in (**e**) dry air and (**f**) air with a relative humidity of 60%.

Therefore, to achieve a high sensitivity in an ultrasonic H_2_ sensor, several conditions must be satisfied. First, the sensitive layer possesses strong sorption to H_2_. Second, the central frequency of the sensor is shifted to the same direction by the mass load and conductivity variation. Since a mass load always decreases the central frequency of a sensor, the conductivity should be increased by the sorbed gas to decrease the central frequency. The abovementioned two conditions can be satisfied using the RGO/Pt sensitive layer because it was proven that the conductivity of a graphene-like film was increased by H_2_^28,30^. Finally, because the central frequency of in an ultrasonic H_2_ sensor is primarily influenced by the variation of the conductivity, the electric response of the sensor must be enhanced to achieve a high sensitivity. Noting that a strong electric response, creating a large frequency shift, can merely be achieved within a narrow conductivity window^42–44^. Thus, we strictly control the initial conductivity of our RGO/Pt sensitive layer in the sensor. The relation between the velocity of the surface acoustic wave and the conductivity of the sensitive layer is calculated using transfer matrix method, which is shown in Fig. 4a,b. Figure 4a shows that in our sensor based on a LiNbO_3_ piezoelectric substrate, the conductivity window for a high conductivity sensitivity is centered at ${\sigma_m} = 1.6\;\mu S$, with a width from ${\sigma^- } = {10^{ - 1}}{\sigma_m}$ to ${\sigma^+ } = {10^{0.5}}{\sigma_m}$. Furthermore, as shown in Fig. 4b, in the left part of the window, the slope of the curve is larger, indicating a higher conductivity sensitivity. Therefore, the initial conductivity of our RGO/Pt sensitive layer is established at ${\sigma_0} = 0.77\;\mu S$, which is located within the window, as shown in Fig. 4b. In this case, a large frequency shift can be induced by the variation of the surface conductivity in the sensor, which adds to the frequency shift originating from the mass load of the adsorbed H_2_. This coaction of two mechanisms, mass load and conductivity variation, can be adopted in any other ultrasonic sensors for detecting matters with trace concentrations to increase the sensitivities.

Moreover, to observe electric turbulence induced by the adsorbed H_2_, we measure the conductivity of the RGO/Pt sensitivity layer using a RTS-8 type 4-probes resistivity measurement system. Figure 4c–f show the relative variation of the surface conductivity of the sensor ${C_r} = {{\sigma_A} \mathord{\left/ {\vphantom {{\sigma_A} {\sigma_0}}} \right. \kern-\nulldelimiterspace} {\sigma_0}}$, which is defined by the conductivity before (${\sigma_0}$) and after (${\sigma_A}$) exposure to H_2_. It can be observed that when H_2_ is introduced into the test box, the conductivity of the sensitive layer increases from its original value ${\sigma_0} = 0.77\;{\mu S}$, resulting in ${\sigma_A} > {\sigma_0}$ and ${C_r} > 1$. It was presented that a increase of the surface conductivity results in a decrease of the central frequency of an ultrasonic sensor^42^. Thus, the central frequency of our sensor is decreased by both the mass load and variation of surface conductivity, which leads to an extremely high sensitivity.

As shown in Fig. 4c,d, the conductivity of the sample is shifted to 4.63 and 4.21 times after exposure in 100 ppm H_2_ mixed in dry argon and argon with a humidity of 60%, respectively. For a recovery process of 10 min, ${C_A}$ returns to $1.52{C_0}$ in dry argon and to $1.34{C_0}$ in wet argon, which indicates a better recovery in a humid environment. Furthermore, as shown in Fig. 4e,f, in the background gas of air, we obtain ${C_r} = 1.72$ and 1.31 in 1000 ppm H_2_ mixed in dry and wet air, respectively, which exhibits that the conductivity variation is smaller in air than that obtained in argon. Meanwhile, comparing Fig. 4e,f shows that the recovery of the sensor in wet air is faster and better than that in dry air. Generally, as shown Figs. 3 and 4, the influences of the background gas and humidity on the conductivity variation resemble to their influences on the frequency shift, which demonstrates the importance of electric turbulence in the response of our H_2_ sensor.

**Figure 5:**
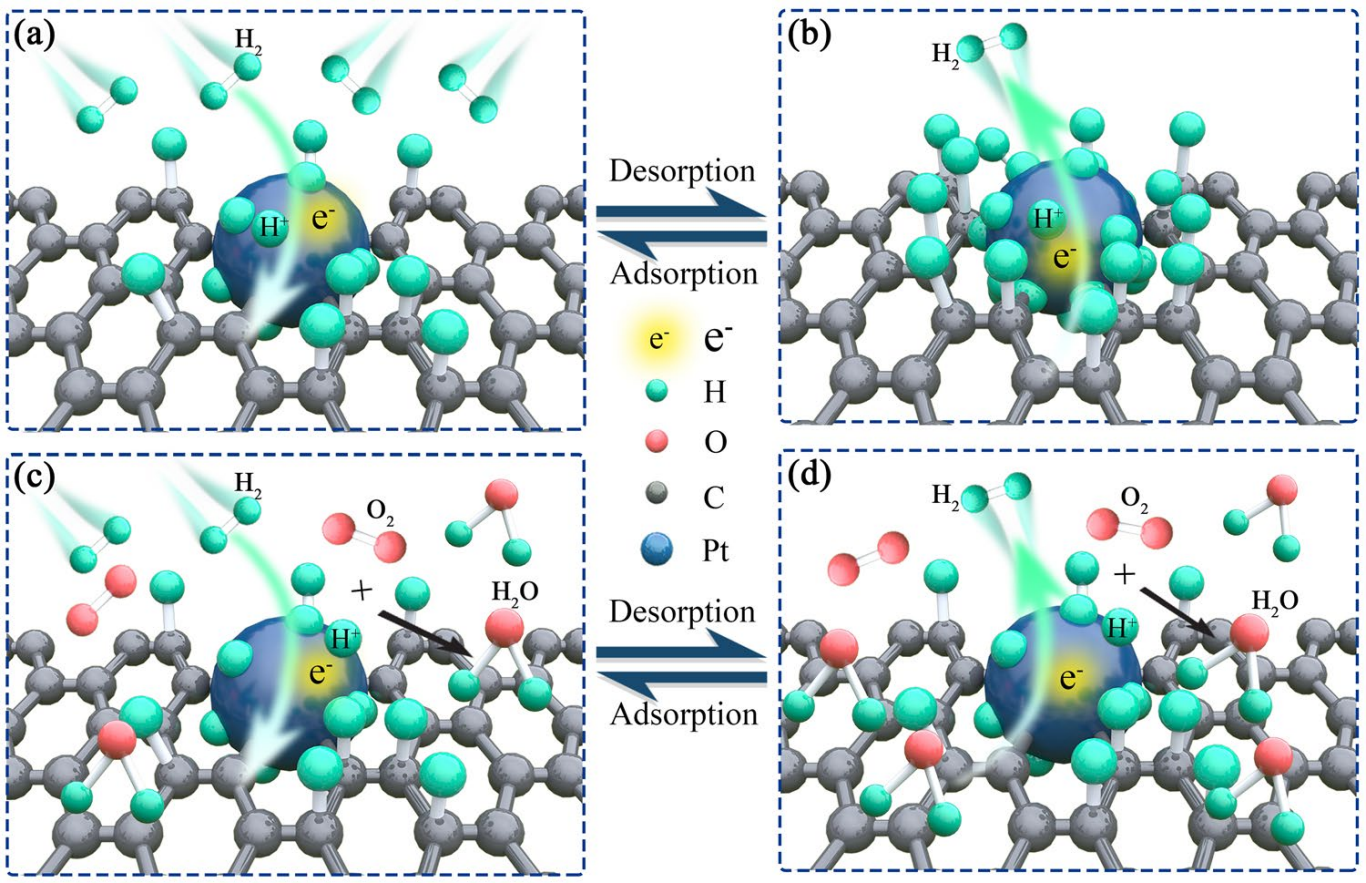
Sensing mechanism. (**a**) Adsorption and (**b**) desorption processes of H_2_ mixed in argon. (**c**) Adsorption and (**d**) desorption processes of H_2_ mixed in air.

The performances of the sensor in different background gases and humidity conditions can be explained on the basis of the sensing mechanism. As shown in Fig. 5a, in the adsorption process, H_2_ molecule is dissociated on the sensitive layer on the basis of competing mechanisms^28,29^, physisorption in which H_2_ is bounded on the surface of Pt by weak van der Waals forces, and chemisorption in which H_2_ enters the lattices of Pt, creating platinum hydride Pt/H and changing the work function of Pt^28^. This process is determined by the sticking coefficient of the platinum catalyst for H_2_ adsorption^46^. Additionally, H_2_ atoms also diffuse through the Pt thin layer^30^, and the concentration of free carriers in RGO will increase, thus improving the conductivity of RGO/Pt sensitive layer^33^.

As indicated in Fig. 5c, with the presence of oxygen, the dissociated H_2_ atoms react with oxygen and form water^34,40^. Then, the concentration of H_2_ bounded by the sensitive layer decreases. Moreover, it was found that water decreases the conductivity of the RGO/Pt layer^47^. Thus, the sensor exhibits a lower sensitivity in air than that in argon.

In the desorption section shown in Fig. 5b, when H_2_ is exhausted, desorption automatically happens for physically adsorbed H_2_ atoms, while the chemically bounded H_2_ cannot be released easily, and thus, the sensor do not return to the original state. Nevertheless, in wet air, as shown in Fig. 5d, the bounded H_2_ atoms react with oxygen in air, creating water molecules. In a high humidity condition, numerous water molecules stay on the surface of the sensitive layer after the desorption of H_2_, further decreasing the surface conductivity, which improves the recovery of the sensor.

**Figure 6:**
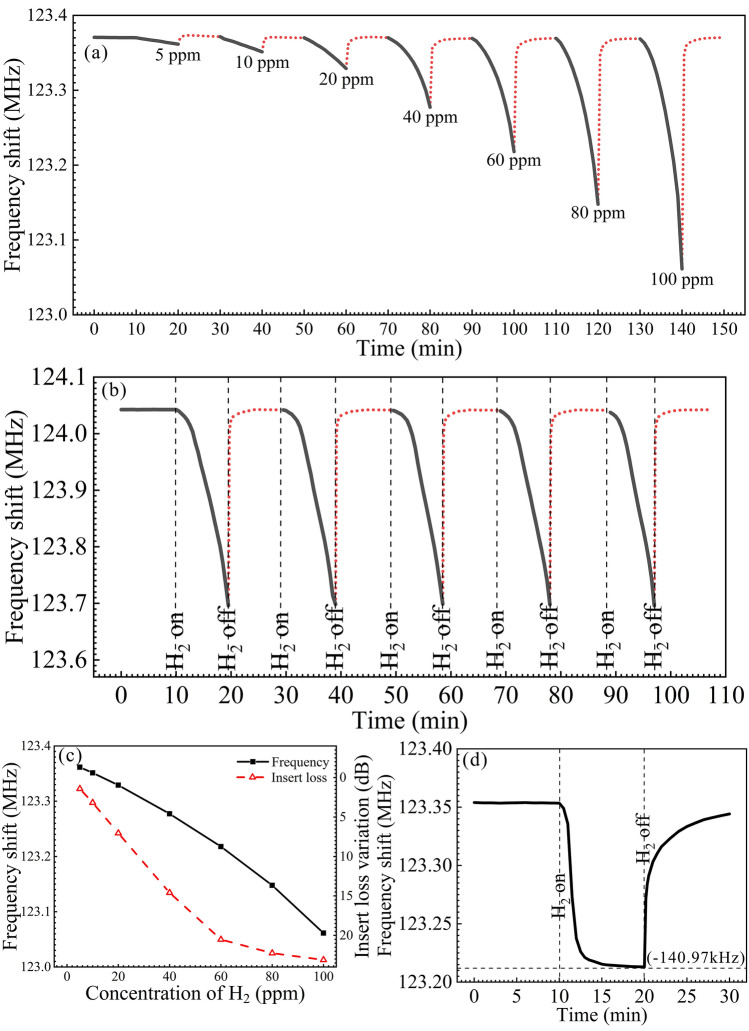
Response of the sensor to H_2_ with different concentrations and NH_3_. (**a**) Dynamic responses of the sensor to H_2_ with different concentrations mixed in argon. (**b**) Dynamic responses of the sensor to five cycles of H_2_ of 100 ppm mixed in argon. To speed up the recovery process, we introduce wet air into the text box to exhaust H_2_ in the experiments. (**c**) The frequency shift and loss variation versus the concentration of H_2_. (**d**) The frequency shift of the sensor to 100 ppm NH_3_ mixed in argon with a relative humidity of 60%.

Finally, we use our sensor to detect H_2_ with different concentrations from 5 to 100 ppm mixed in argon. To speed up the recovery process, we inlet wet air into the testing box in the desorption section. The dynamic response of the sensor to H_2_ with different concentrations is shown in Fig. 6a. The sensor exhibits a frequency shift of 8.7 kHz towards H_2_ with a concentration of 5 ppm, which demonstrates that the sensor is efficient for detecting H_2_ with an extremely low concentration. Moreover, by introducing wet air in the recovery section, the central frequency of the sensor rapidly return to the original value. It can be observed that the sensor responses swiftly to H_2_ with low concentrations. Additionally, we measure the response of our sensor to five cycles of H_2_ flow with a concentration of 100 ppm. As shown in Fig. 6b, the sensor can stably response and recover in the repeated progresses. Furthermore, the insert loss of the sensor is also measured. The frequency shift and insert loss variation measured in H_2_ with different concentrations are shown in Fig. 6c which show similar tendencies. Finally, we use our sensor to detect ammonia gas (NH_3_) with a concentration of 100 ppm mixed in argon with a relative humidity of 60%. As shown in Fig. 6d, it can be seen that the central frequency of the sensor decreases by 140.97 kHz in 10 min, which is lower than the frequency shift to H_2_. Moreover, it is observed that in NH_3_, the frequency of the sensor marginally changes after 5 min, while in H_2_, the frequency continues to decrease after 10 min, which demonstrates that our sensor is more sensitive to H_2_ than to NH_3_.

In conclusion, we present an ultrasonic H_2_ sensor on the basis of a LiNbO_3_ piezoelectric waveguide and a RGO/Pt sensitive layer. RGO solution is prepared using hydrothermal reduction method, and a sedimentation method is adopted to efficiently create multiple samples of the H_2_ sensors. The images of XRD, FTIR and SEM exhibit that the sensitive layer possesses small grains, high porosities and large specific surface areas, which provide strong adsorbability of H_2_. In the experiments of H_2_ sensing, our sensor exhibits an extremely high sensitivity, which is 308.9 kHz towards 100 ppm H_2_ mixed in argon and 24.1 kHz towards 1000 ppm H_2_ mixed in air. In addition to the high performance of the RGO/Pt sensitive layer in H_2_ adsorption, the high sensitivity is primarily achieved owing to the coaction of the mass load and conductivity variation induced by the adsorbed H_2_. By accurately controlling the initial surface conductivity within the window for strong electric response, the conductivity variation greatly decreases the central frequency, which adds to the frequency shift induced by the mass load and considerably increases the sensitivity of the sensor. Additionally, it is found that oxygen and water molecules speed up the recovery process of the sensor. Thus, our sensor swiftly responses to trace concentrations of H_2_ and the sensor can be available to detect H_2_ with a low concentration below 5 ppm. Furthermore, the synergistic effect of two mechanisms, mass load and conductivity variation, adopted in this H_2_ sensor can be applied in any other ultrasonic sensors for enhancing the performance in detection of matters with trace concentrations.

## Methods

### RGO preparation

First, graphite powder (325 mesh) and sodium nitrate are stirred into refrigerated concentrated sulfuric acid in an ice bath. Then, potassium permanganate is added into the suspension, which continues to be stirred in the ice bath. The temperature of the suspension is increased to 35 °C and maintained for 30 min. Deionized water is added into the suspension and the temperature increases to over 90 °C. The mixture is kept in a water bath at a temperature of 90 °C for 20 min. The suspension is then diluted with warm deionized water and treated using hydrogen peroxide to eliminate the residual potassium permanganate. The treated suspension is filtered and the filter cake is washed in a centrifuge with hydrochloric acid (4%) and deionized water until the supernatant becomes neutral, and we obtain a suspension of GO. The suspension is dialyzed for two days in a dialysis bag with a molecular weight cut-off of 8000–14,000 and the remained solid is sufficiently dried at 50 °C in a vacuum drying oven. Then, GO sheets are obtained, which are grinded into powder in a agate mortar. The powder is added into deionized water and the suspension is exfoliated in an ultrasonic cleaner for 30 min and in an ultrasonic cell disruptor for 30 min under the condition of ice bath. Next, the exfoliated GO is deoxidized using hydrothermal reduction method. The GO solution is diluted to 0.2 mg/ml and transferred to an autoclave. The solution is heated at 180 °C for 6 h, and then we obtain a solution of RGO. Additionally, to study influence of the reduction degree on the performance of the RGO, we conducted hydrothermal reduction at a different reaction temperature of 120 °C.

### Sensitive layer deposition

First, interdigital transducers with a period width of 32 μm is created on a LiNbO_3_ substrate with lithographic technology. Then, we use a sedimentation method to deposit RGO sensitive layer on the surface of the LiNbO_3_ substrate. The concentration of the solution is strictly controlled. Generally, using a solution with a high concentration, the deposited sensitive layer is thicker and smoother, while a thick sensitive layer increases the loss of the sensor. Therefore, we dilute the RGO solution to ${0}{\text{.175}}\;{{{\text{mg}}} \mathord{\left/ {\vphantom {{{\text{mg}}} {{\text{ml}}}}} \right. \kern-\nulldelimiterspace} {{\text{ml}}}}$ and drop the solution (120 μl) on the LiNbO_3_ substrate. The sample is dried for over 12 h at room temperature, and thus, a RGO sensitive layer is achieved. Finally, magnetron sputtering method is used to grow Pt on the surface of the sample. This process is critical to high sensitivity of our sensor. Pt is used as a catalyzer, and moreover, the conductivity of the sensitive layer is also influenced by the Pt layer. We use a sputtering power of 15 W and a sputtering time of 3 s. As a result, the conductivity of our sensitive layer is established to be $\sigma = 0.77 \mu S$, which accurately locates within the narrow window for a high conductivity sensitivity.
